# Factors influencing the positivity of diagnostic tests for congenital syphilis

**DOI:** 10.1590/1806-9282.20231006

**Published:** 2024-04-22

**Authors:** Rodrigo Soares Ribeiro, Natália Sperli Geraldes Marin dos Santos Sasaki, Alessandra Marinela de Abreu Queiroz, Ana Cecília Mota Ferreira, Gabriela de Souza Segura, Maria de Lourdes Sperli Geraldes Santos, Lara Helk de Souza, Luciano Garcia Lourenção

**Affiliations:** 1 Faculty of Medicine of São José do Rio Preto - São José do Rio Preto (SP), Brazil.; 2 Union of Colleges of Great Lakes - São José do Rio Preto (SP), Brazil.; 3 Universidade Federal do Rio Grande - Rio Grande (RS), Brazil.

**Keywords:** Syphilis, Congenital, Early diagnosis, Prenatal care

## Abstract

**OBJECTIVE:**

The objective of this study was to analyze the factors that influence the positivity of treponemal and non-treponemal tests in cases of congenital syphilis.

**METHODS:**

This cross-sectional and correlational study was carried out from the analysis of the database of Disease and Notification Information System (SINAN, in Portuguese) using the data obtained through the Epidemiological Surveillance Group 29, with 639 notifications of congenital syphilis between 2007 and 2018. The data were analyzed by a descriptive and inferential analysis from logistic regression with a significance level of 5% (p≤0.05).

**RESULTS:**

The positivity of the treponemal test was higher by 4.5 times in infants living in rural areas and 19.6 times among those whose mothers obtained the diagnosis of syphilis after birth. The treponemal test showed positivity 3.2 times higher for the variable “having been diagnosed between 2007 and 2015” and 5.5 times higher for the variable “having been diagnosed with maternal syphilis in the postpartum period.”

**CONCLUSION:**

This study shows that testing during prenatal care is essential for early diagnosis and prevention of syphilis complications.

## INTRODUCTION

Vertical transmission of syphilis can lead to serious fetal problems such as miscarriage, prematurity, and death, in addition to congenital infections^1^ ^,^ ^2^. Prevention involves early detection and treatment during pregnancy. If the pregnant woman receives proper treatment, the fetus is easily cured and its adverse effects are minimized, especially before the third gestational trimester. Evidence shows a worldwide decline in mother-to-child transmission of syphilis, due to advances in screening and early detection of infection^1^ ^,^ ^3^.

Prenatal syphilis screening by rapid testing has been a strategy of the World Health Organization (WHO) to improve timely detection and adequate treatment to interrupt vertical transmission. The rapid test does not require laboratory infrastructure and can be easily taken to people in rural areas or those who do not have easily accessible health services. They are simple and affordable tests, making them useful in all types of care^1^ ^,^ ^3^ ^,^ ^4^. Despite this, there are barriers such as logistics, infrastructure, professional training, and lack of knowledge among pregnant women about the applicability of rapid tests^5^.

In Brazil, women’s access to primary health care units is not equal, due to inadequate infrastructure (difficult access, lack of rooms, use of temporary spaces), disproportionate coverage between regions, and restricted opening hours (limited to business hours), as many women are unable to leave work to go to the health service^6^.

In view of the above, this study aimed to analyze the factors that influence the positivity of treponemal and non-treponemal tests in cases of congenital syphilis.

## METHODS

### Study design, period, and location

This cross-sectional, descriptive, and correlational study was carried out between January and March 2019, using the data from the Disease and Notification Information System (SINAN, in Portuguese), obtained through Epidemiological Surveillance Group 29, a regional member of the structure of the “Prof Alexandre Vranjac” Epidemiological Surveillance Center (CVE/SP), which regulates the Epidemiological Surveillance System in the State of São Paulo. This research was guided by the Strengthening the Reporting of Observational Studies in Epidemiology (STROBE) tool.

The Epidemiological Surveillance Group 29 covers 67 municipalities belonging to the Regional Management Collegiate of Catanduva, José Bonifácio, Votuporanga, and São José do Rio Preto, where it is headquartered.

### Population and sample

The study included the congenital syphilis cases notified by SINAN in the municipalities integrating the Epidemiological Surveillance Group 29, in the period from January 2007 to December 2018. This period was defined in agreement with the GVE coordination, taking into account the quality and completeness of the data available in the system and notification. The rapid test for diagnosis was introduced in 2016.

### Study protocol

The variables explored included information from the child, pregnant woman, and sexual partner which were considered as independent variables. Regarding children, the variables comprised general data and individual notification as the period of notification according to the change in diagnostic guidelines (2007-2015 and 2016-2018)^7^, area of residence, sex, color, and death. Regarding pregnant women, socioeconomic variables were analyzed (maternal age group according to the classification considered with gestational risk, color, and education in years of schooling), clinical and laboratory variables (prenatal care, diagnosis of maternal syphilis, and title of the non-treponemal test), and concomitant treatment of the partner. For the non-treponemal test titer, the reference values used were greater than 1:8 or less than or equal to 1:8, because false-positive results generally have titers less than 1:84. The dependent variables were the reactivity of the non-treponemal test and the treponemal test.

### Analysis of results and statistics

Data analysis was performed using the software Statistical Package for the Social Sciences (SPSS), version 20.0. Initially, a univariate analysis was performed with the calculation of Pearson’s or Fischer’s chi-square tests when necessary, considering a significance level of 5% (p≤0.05). Ignored or blank cases were excluded as they appeared in the data cross-analyses. Subsequently, a multivariate analysis was performed using stepwise binary logistic regression in which the variables that obtained p≤0.20 in the univariate analysis and that did not present multicollinearity were included. The data were presented in contingency tables.

### Ethical aspects

The research complies with the ethical precepts and was approved by the Research Ethics Committee under opinion 2.556.704 and CAAE: 85159518.0.0000.5489.

## RESULTS

During the study period, there were 639 notifications of congenital syphilis, of which 92.8% had a reagent non-treponemal test and 78.2% had a reagent treponemal test. There was statistical significance between the non-treponemal test only with a zone of residence (p=0.015) and the treponemal test with a period of notification (p=0.001) and stillbirth, neonatal, and infant death (p=0.050). Among maternal variables, there was statistical significance between treponemal and non-treponemal tests related only to maternal syphilis diagnosis (p<0.001) (Table 1).

**Table 1.** Results of treponemal and non-treponemal tests according to sociodemographic, clinical, and treatment variables of the mother and child and treatment of the partner (São Paulo, Brazil).

Variables (n=639)	Non-treponemal test		Treponemal tests	
	Non-reactive	Reactive	Non-reactive	Reactive
	n (%)	n (%)	n (%)	n (%)
	46 (7.2)	593 (92.8)	139 (21.8)	500 (78.2)
Reporting period	p=0.075		p=0.001	
2007-2015	34 (8.4)	369 (91.6)	104 (25.8)	299 (74.2)
2016-2018	12 (5.1)	224 (94.9)	35 (14.8)	201 (85.2)
Living area*	p=0.015		p=0.102	
Urban	37 (6.1)	566 (93.9)	128 (21.2)	475(78.8)
Rural	18 (78.3)	5 (21.7)	8 (34.8)	15 (65.2)
Gender of the child*	p=0.358		p=0.413	
Female	21 (6.8)	286 (93.2)	69 (22.5)	238 (77.5)
Male	22 (8.0)	254 (92.0)	59 (21.4)	217(78.6)
Child’s skin color*	p=0.529		p=0.164	
White	34 (7.0)	451 (93.0)	100 (20.6)	385 (79.4)
Other	6 (6.5)	87(93.5)	24(25.8)	69 (74.2)
Stillbirth, neonatal, and infant death	p=0.501		p=0.050	
Yes	6 (7.7)	72 (92.3)	11 (14.1)	67 (85.9)
No	40 (7.1)	521 (92.9)	128 (22.8)	433 (77.2)
Maternal age group*	p=0.280		p=0.664	
<18 years	2 (3.8)	50 (96.2)	12 (23.1)	40 (74.9)
18-34 years	42 (7.9)	493 (92.1)	115 (21.5)	420 (78.5)
35 years or older	1 (2.5)	39 (97.5)	11 (27.5)	29 (72.5)
Mother’s skin color*	p=0.248		p=0.409	
White	25 (6.0)	395 (94.0)	90 (21.4)	330 (78.6)
Other	15 (7.8)	178 (92.2)	39 (20.2)	154 (79.8)
Mother’s education (in years)*	p=0.553		p=0.108	
Up to 8 years	15 (6.8)	204 (93.2)	53 (24.2)	166 (75.8)
9 years or more	19 (6.8)	262 (93.2)	54 (19.2)	227(80.8)
Prenatal*	p=0.541		p=0.527	
Yes	39 (7.0)	519 (93.0)	120 (21.5)	438 (78.5)
No	5 (6.4)	73 (93.6)	17 (21.8)	61 (78.2)
Maternal syphilis diagnosis*	p<0.001		p<0.001	
Postpartum	12 (54.5)	10 (45.5)	14 (63.6)	8 (36.4)
During delivery	3 (1.7)	177 (98.3)	36 (20.0)	144 (80.0)
Prenatal	26 (6.1)	402 (93.9)	83 (19.4)	345 (80.6)
Non-treponemal test title*	p=0.405		p=0.544	
≤1:8	2 (0.5)	377 (99.5)	72 (19.0)	307 (81.0)
>1:8	0 (0.0)	216 (100.0)	41 (19.0)	175 (81.0)
Treatment scheme	p=0.127		p=0.368	
Adequate	0 (0.0)	27 (100.0)	7 (25.9)	20 (74.1)
Inadequate	46 (7.5)	566 (92.5)	132 (21.6)	480 (78.4)
Concomitant partner treatment	p=0.405		p=0.190	
Yes	8 (6.2)	120 (93.8)	32 (25.0)	96 (75.0)
No	38 (7.4)	473 (92.6)	107 (20.9)	404 (79.1)

*Exclusion of cases with ignored information.

The results showed that 92.5% of non-treponemal test-positive cases followed an inadequate treatment regimen. This practice was observed in 78.4% of the treponemal test-positive cases (Table 1). The concomitant treatment of the partner was not carried out in 92.6% of the non-treponemal test-positive cases and 79.1% of the treponemal test-positive cases (Table 1).

For logistic regression, only variables with p≤0.05 were included in the step-wise analysis (Table 2). The variables “area of residence” and “maternal diagnosis of syphilis” were included in the model for the positivity of the non-treponemal test. The period of diagnosis, concomitant partner testing, and maternal syphilis diagnosis were included in the model for the positivity of the treponemal test. Living in a rural area and being diagnosed in the postpartum period represented, respectively, 4.5 and 19.6 times greater chances of being positive for the non-treponemal test. On the contrary, the chances of being positive for the treponemal test were 3.2 times higher for the variable “having been diagnosed between 2007 and 2015” and 5.5 times higher for the variable “having been diagnosed with maternal syphilis in the postpartum period.” Newborn deaths were five times higher among those who tested positive. Diagnosis at delivery was a protective factor for positivity in both tests (Table 2).

**Table 2.** Logistic regression analysis between the reacting results of non-treponemal and treponemal tests (São Paulo, Brazil).

Variables			OR	95%CI	p-value
Non-treponemal test	Living area	Rural	4.585	1.388-15.147	0.013
		Urban	1		
	Maternal syphilis diagnosis	Prenatal	1		
		during the birth	0.290	0.086-0.979	0.046
		postpartum	19.619	7.579-50.783	<0.001
Treponemal tests	Reporting period	2007-2015	3.176	1.780-5.667	<0.001
		2016-2018	1		
	Concomitant partner treatment	Yes	1		
		No	0.426	0.245-0.742	0.003
	Maternal syphilis diagnosis	Postpartum	5.573	1.737-17.881	0.004
		During delivery	1.003	0.557-1.774	0.991
		Prenatal	1		

OR: odds ratio; CI: confidence interval.

## DISCUSSION

This study showed that the positivity of the non-treponemal test was influenced by the area of residence and maternal diagnosis after delivery, while the cases with syphilis maternal diagnosis at delivery had a protective factor. As for the treponemal test, besides the three previous factors, death was also a predictor of greater chances of reactivity. The protective factor related to syphilis diagnosis at the time of delivery is related to the identification of treponema before the disease is activated in the newborn and without complications for the mother^8^. This is why it is essential to carry out the rapid test at the time of delivery, even if the pregnant woman has already been tested in the last trimester of pregnancy.

In this regard, it is important to note that the strengthening of primary health care services, by expanding the coverage of the family health strategy and building attention networks, brought greater qualification of care, expanding the monitoring through the assignment of the territory and linkage with the team and making the diagnosis of diseases more accessible to the population^6^ ^,^ ^9^. However, some challenges are still present, such as the high percentage of cases with inadequate treatment regimens and the low rate of concomitant treatment of the partner, observed in this study and reported in the literature^8^.

Syphilis diagnosis is based on direct and immunological tests. Immunological tests are most commonly used in clinical practice and are divided into non-treponemal and treponemal tests. The non-treponemal tests detect the non-specific anticardiolipin of the *Treponema pallidum* antigen that allows quali-quantitative analysis and the result is expressed in progressive fraction, allowing monitoring of the therapeutic response or evolution of the infection. However, late or latent infection has low titers^9^. The treponemal tests detect specific antibodies produced against the *T. pallidum* antigens, which are the first post-infection tests to present reagent results and, in approximately 85% of infected persons, remain reagent for life, thus requiring non-treponemal tests to evaluate the therapeutic response^4^.

Rapid treponemal tests are quick to perform, read, and interpret. Performed with a small amount of blood in digital or venipuncture, serum, or plasma, they do not require laboratory structure, ensuring an improvement in screening and early diagnosis^3^ ^,^ ^4^. The Ministry of Health recommends its use in pregnant women with previous contact to the disease, who may develop a high risk of untreated syphilis. False-negative results may occur in the initial phase of the disease, requiring the association of a treponemal test with a non-treponemal test^10^.

Testing for syphilis is recommended during the first prenatal visit, from the 28th week of gestation, at the time of birth, or in cases of abortion, regardless of having been previously tested^4^. The expansion of rapid testing during prenatal care has contributed to the identification of asymptomatic pregnant women and caused a significant increase in cases of acquired, gestational, and congenital syphilis^11^.

Early initiation of prenatal care is essential for preventing the development of congenital syphilis. To this end, the access of pregnant women to health services should be expanded, to ensure the prevention of complications during pregnancy and to the conceptus^2^ ^,^ ^6^ ^,^ ^12^ ^,^ ^13^ ^,^ ^14^ ^,^ ^15^ ^,^ ^16^.

Access to health services is addressed by the concept of accessibility, which involves aspects of how people enter the health care network and how professional and technological resources are organized to serve them^6^. Accessibility encompasses the organizational, sociocultural, economic, and geographic dimensions. Despite the investments provided by the National Program for Improving Access and Quality of Primary Care (PMAQ-AB), regional disparities in access and accessibility to primary care services in Brazil are still observed, mainly related to the infrastructure of services, which require investment to improve access. These failures cause major difficulties in achieving resoluteness for the population’s health problems^6^ ^,^ ^12^ ^,^ ^16^.

Neglected diseases, such as syphilis, are associated with socioeconomic conditions and people living in poverty, which generate an important condition of vulnerability for the population exposed to the risk of contamination. To overcome this condition, it is important to implement health actions and policies that improve people’s knowledge about the problem, arousing interest and creating possibilities to transform concerns into protective practices, including the search for early diagnosis^17^ ^,^ ^18^.

Another crucial factor for the prevention of congenital syphilis is the treatment with benzathine penicillin G, which is considered the first option (gold standard) for the treatment of syphilis in pregnancy. In 2014, there was a shortage due to a worldwide shortage of the drug, and it was re-established the following year^19^ ^,^ ^20^ ^,^ ^21^. Although the drug shortage occurred in almost all the studied municipalities, in one of them, this did not occur due to the organization of the health system, with a provision in the previous year and the structuring of clinical protocols for the detection and control of syphilis. Therefore, health planning is important for the success of policies for diagnosis and control of communicable diseases such as syphilis, which contributes to organize the entire structure of services and standardization of treatments^11^.

In this aspect, one can suggest weaknesses in prenatal care with respect to screening for *T. pallidum* infection in the three moments of prenatal care, highlighting the importance of screening at delivery by rapid test^4^ ^,^ ^22^.

At delivery, the maternal diagnosis of syphilis allows the mother and her partner to be treated, avoiding complications. However, at this time, it will not be able to prevent transmission of the disease to the baby; it is no longer timely. Even so, the diagnosis of gestational syphilis at the time of birth offers the possibility of treating the baby, avoiding the severe consequences of late Congenital Syphilis, such as neurosyphilis^4^ ^,^ ^15^.

For years, syphilis has been diagnosed by means of the VDRL test, which is considered simple and of low-cost, but it requires a laboratory structure for its performance. The late initiation of prenatal care associated with delayed results when returning to the clinic may contribute to late access to VDRL results during prenatal care^4^.

When titers decrease around two dilutions in 3 months, non-treponemal tests indicate treatment success, such as a result that was 1:64 and dropped to 1:16. Persistent low titers after 1 year of treatment, if there is no possibility of a new infection in this period, is also considered a successful treatment. Persistent low titers indicate serological scarring and may last for a lifetime. However, if the titer is elevated by two dilutions or more, the possibility of reinfection or reactivation of the infection should be considered, requiring drug treatment^4^ ^,^ ^14^.

The reinfection of pregnant women by syphilis is also associated with the non-treatment of their sexual partners and the consequent increase in vertical transmission. The unfavorable outcomes for newborns with congenital syphilis are independent of the treatment of the pregnant woman’s sexual partner, considering that the syphilis infection was late in pregnancy^1^ ^,^ ^15^. The Ministry of Health recommends that, regardless of the syphilis stage diagnosed in the pregnant woman, all sexual partners exposed in the last 90 days before the diagnosis of gestational syphilis should be treated. This extends to sexual partners of contact greater than 90 days and those who had intercourse in the latent phase should be clinically evaluated^4^.

For the treatment of sexual partners of pregnant women, it should be assumed that they are infected, even with non-reactive immunological tests. Therefore, they should presumably be treated with only one dose of intramuscular benzathine penicillin. In case of a reactive test for syphilis, one should follow the recommendations for the treatment of adult-acquired syphilis, according to the clinical stage of infection, preferably using benzathine penicillin^4^ ^,^ ^15^.

The study has some limitations, such as the quality of the SINAN database, which generated incomplete data^23^, and geographical delimitation, which includes cultural factors that restrict the generalization of the results. However, the results show that congenital syphilis remains a major challenge to public health, stressing that the characteristics linked to the binomial regarding health care point to low effectiveness in prenatal care regarding the appropriate treatment, interruption of vertical transmission, and treatment of sexual partners of mothers infected with *T. pallidum*. These results highlight the need to strengthen public policies aimed at diagnosing congenital syphilis and call for new studies covering other regions of the country.

## CONCLUSION

This study showed that living in rural areas, maternal diagnosis of syphilis after birth contributes to a greater chance of having a positive non-treponemal and treponemal test, and the death of the newborn increases the positivity of the treponemal test. Rapid testing in maternity hospitals proved to be effective in detecting the disease.
